# Tough Hydrogel Reinforced by Meta-Aramid Nanofibers for Flexible Sensors

**DOI:** 10.3390/polym17162179

**Published:** 2025-08-09

**Authors:** Zhiwen Hou, Yongzheng Li, Donghao Zhang, Cun Peng, Yan Wang, Kunyan Sui

**Affiliations:** 1 Key Laboratory of Marine Bio-Based Fibers of Shandong Province, Key Laboratory of Shandong Provincial Universities for Advanced Fibers and Composites, Qingdao Application Technology Innovation Center of Advanced Fibers and Composites, College of Materials Science and Engineering, Qingdao University, Qingdao 266071, China; 15216380929@163.com (Z.H.);; 2 College of Chemistry and Chemical Engineering, Qingdao University, Qingdao 266071, China

**Keywords:** hydrogels, aramid nanofibers, mechanical reinforcement, wearable sensors

## Abstract

Hydrogels exhibit significant promise for advanced flexible sensing applications owing to their intrinsic softness, biocompatibility, and customizable functionalities. Nevertheless, their limited mechanical strength poses a critical barrier to practical implementation. In this study, we engineered a mechanically robust alginate/chitosan (SA/CS) hydrogel reinforced with meta-aramid (PMIA) nanofibers. The resulting composite hydrogel achieves a tensile strength of 16.8 MPa, substantially exceeding the performance of conventional biomass-derived hydrogels. When employed as a flexible sensor, the hydrogel demonstrates exceptional pressure-sensing capabilities, featuring high sensitivity (178.41 MΩ/MPa below 5 kPa), rapid response kinetics (0.4–0.8 s), and sustained stability (>200 cycles). Leveraging these properties, we successfully monitored vocal cord vibrations and finger motion trajectories, highlighting their potential for biomechanical sensing applications.

## 1. Introduction

Hydrogel materials have demonstrated immense application potential in numerous fields such as wearable devices, flexible energy storage devices, biomedical engineering, and soft robotics, making them a current research hotspot [1,2,3]. Conventional hydrogels often lack sufficient toughness due to ineffective energy dissipation mechanisms. To date, researchers have proposed various strategies—such as employing chemical crosslinking, introducing reversible sacrificial bonds, and incorporating nanocomposites—to enhance the energy dissipation mechanisms of hydrogels [4,5,6]. A variety of high-performance, multifunctional hydrogel materials have been developed, endowing hydrogel-based devices with functionalities/capabilities akin to biological systems, including ion gating, self-healing, damping, ultra-stretchability, and fatigue resistance [7,8,9]. However, current high-performance gel materials often require complex preparation methods, and achieving both a high-strength and simple process remains a critical challenge in this field.

Natural polysaccharides offer advantages such as non-toxicity, renewability, abundance, low cost, and biodegradability. Polysaccharide-based hydrogels have thus attracted widespread attention and are considered one of the most promising alternatives to synthetic polymers. Nevertheless, natural polysaccharide hydrogels generally exhibit poor mechanical properties [10,11,12]. In previous studies, our team innovatively proposed a diffusion–complexation strategy for synthesizing high-strength all-natural polysaccharide polyelectrolyte gels, which enables the rapid and large-scale fabrication of polysaccharide hydrogels [13,14]. Although the strength of the synthesized gels can reach 1–3 MPa, it still falls far short of that achieved by some synthetic hydrogel materials. There is an urgent need to develop novel strategies for constructing natural hydrogels to expand their applications in tissue engineering, flexible wearable sensors, implantable devices, and other fields [15,16]. In recent years, the nanofiller reinforcement strategy has provided novel insights to address this challenge. By incorporating nanofillers such as carbon nanotubes, graphene, nanocellulose, or inorganic nanoparticles into polymer networks, the mechanical properties of hydrogels (e.g., strength, toughness, and fatigue resistance) can be remarkably enhanced, while simultaneously endowing them with multifunctional characteristics like conductivity, thermal responsiveness, or self-healing capabilities [17,18,19,20,21,22]. Aramid nanofibers amplify the intrinsic advantages of aramid (high temperature resistance, high strength, insulation) through nanoscale effects, and break through the processing limitations, becoming an ideal substrate for a new generation of self-powered wearable electronics, especially in extreme environmental protection fields such as fire protection and military [23].

In this work, by coupling the nanocomposite reinforcement strategy with the diffusion-complexation method, we successfully developed ultra-high-strength meta-aramid (PMIA) nanofiber-reinforced composite hydrogels and demonstrated their applications in flexible wearable sensors and self-deforming actuators. By mixing self-assembled PMIA nanofibers with sodium alginate (SA) polyanion solution and leveraging the diffusion and complexation reaction of low-molecular-weight chitosan (CS) polycations, we synthesized ultra-high-strength PMIA nanofiber-reinforced SA/CS composite hydrogels (PMIA-SA/CS hydrogels). The tensile strength of the resulting natural polysaccharide composite hydrogel reaches an impressive 16.8 MPa, far surpassing that of previously reported biomass hydrogel materials. Although aramid nanofiber reinforcement strategies have been used to improve the mechanical properties of hydrogels, existing studies still have limitations: most of the reinforcement objects are synthetic polymers (e.g., PVA, PAAm), and the enhancement efficiency of natural polysaccharide systems is insufficient (generally < 10 MPa) [24]. The novelty of this paper lies in the fact that the breakthrough strength of 16.8 MPa was achieved for the first time in a pure natural polysaccharide system (SA/CS) through the directional arrangement and diffusion–complexation synergy of aramid nanofibers, which was 124% higher than the literature value, and the high ductility (48% strain) was maintained at a low addition of 1 wt%. This hydrogel material can be used in sensors and self-deforming actuators, showing great promise for applications in flexible wearables, health monitoring, soft robotics, tissue engineering, and beyond.

## 2. Materials and Methods

### 2.1. Materials

Sodium alginate (SA), Mw = 240 kDa, was purchased from Qingdao Haizhilin Biotechnology Development Co., Ltd., Qingdao, China. Chitosan (CS), Mw = 4.5 kDa, deacetylation degree ≥ 90%, was purchased from Weifang Dongxing Shell Products Factory, Weifang, China. Meta-aramid stock solution was purchased from Yantai Tayho Advanced Materials Co., Ltd., Yantai, China. Dimethylacetamide (DMAc) was purchased from Aladdin, Shanghai, China.

### 2.2. Synthesis of Meta-Aramid Nanofibers

To prepare a stable aramid nanofiber suspension, a 30 wt% homogeneous coagulation bath was first prepared by adding an appropriate amount of DMAc to distilled water in a beaker. Under continuous mechanical stirring, a 0.5 wt% aramid solution was then injected into the coagulation bath using a syringe, resulting in the precipitation of aramid nanofibers. The precipitated nanofibers were subsequently collected by centrifugation, thoroughly washed with deionized water, and finally dispersed to form a stable nanofiber suspension.

### 2.3. Synthesis of Meta-Aramid (PMIA) Reinforced SA/CS Composite Hydrogel

First, sodium alginate (SA) powder was added to a meta-aramid nanofiber suspension and magnetically stirred at room temperature for 6 h to obtain a homogeneous SA solution containing PMIA. The SA/PMIA mixture was blade-coated (gap = 2 mm) onto glass without solvent evaporation. Thickness was controlled by a film applicator. Finally, the coated sample was immersed in a chitosan (CS) solution for 6 h to allow complete crosslinking, yielding the final PMIA-reinforced SA/CS composite hydrogel. The formulation of the sample is shown in Table 1.

### 2.4. Characterizations

The morphologies of meta-aramid (PMIA)-reinforced SA/CS composite hydrogel were observed by a field-emission scanning electron microscope (JSM7800F, JEOL Ltd., Tokyo, Japan) operating at 10 kV. The chemical structures of the hydrogel were characterized using an attenuated total reflection mode FTIR (Nicolet IS50, Thermo Fisher Scientific, Madison, WI, USA) and an X-ray photoelectron spectrometer (EscaLab Xi+, Thermo Fisher Scientific, Madison, WI, USA). The mechanical properties were characterized with the hydrogel using a testing machine (WDW-5T, Giroo Precision Machinery Co., Ltd., Shanghai, China) at a compression and tensile speed of 5 mm/min. All hydrogels are tested in a swollen state (24 h in DI water, swelling ratio ~300%).

The resistance test was conducted by connecting the gel through a platinum plate with a digital source meter and applying different pressures to the gel at a constant voltage of 0.1 V.

## 3. Results

### 3.1. Morphologies of Meta-Aramid Nanofibers and Meta-Aramid Nanofiber-Reinforced Composite Hydrogel

**Scheme 1:**
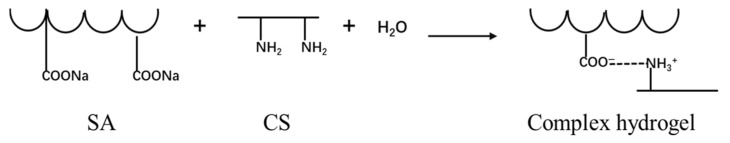
Reaction equation between SA and CS.

**Figure 1:**
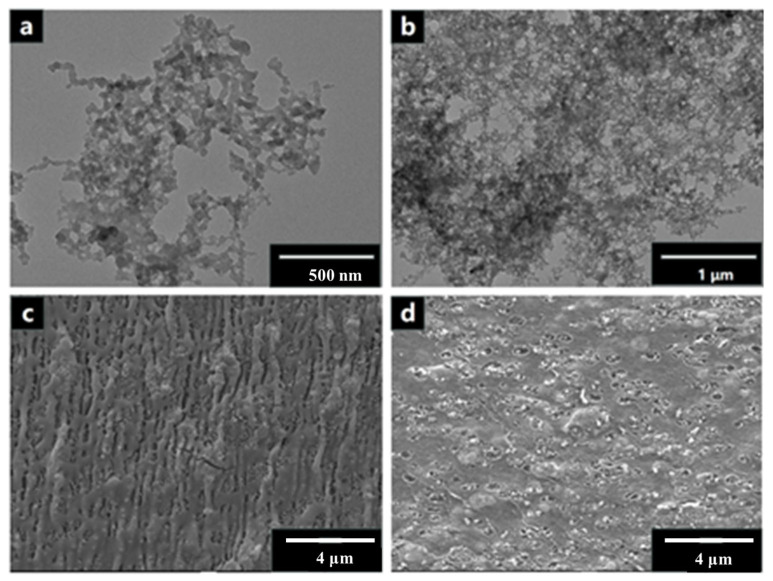
(**a**,**b**) TEM images of meta-aramid nanofibers (PMIA). (**c**) SEM image of the surface of PMIA-SA/CS composite hydrogel. (**d**) Cross-sectional SEM image of PMIA-SA/CS composite hydrogel.

Figure 1a,b shows the TEM image of meta-aramid (PMIA) nanofibers, revealing their characteristic linear fibrous morphology with uniform diameter distribution averaging 20–50 nm. The nanofibers self-assemble into a three-dimensional network structure through van der Waals forces and localized hydrogen bonding interactions (As shown in Scheme 1). This unique nanofiber architecture not only endows the material with a high specific surface area but also provides the structural foundation for its outstanding mechanical properties [23]. When compounded with hydrogel matrices, the meta-aramid nanofiber network can form an interpenetrating network with hydrogel polymers. This structure enhances the interfacial compatibility between the filler and matrix, facilitating efficient stress transfer within the meta-aramid nanofiber network, thereby achieving effective reinforcement of the hydrogel.

The cross-section and surface morphologies of meta-aramid nanofiber-reinforced composite hydrogels were characterized using scanning electron microscopy (SEM), as shown in Figure 1c,d. In the image of surface morphology (Figure 1c), there is a distinct orientation of nanofibers along the coating direction due to the directional deposition process. This aligned microstructure can further enhance the tensile strength of the PMIA-reinforced SA/CS hydrogels. In the image of cross-section (Figure 1d), it can be observed that a uniform distribution of PMIA nanofibers is present throughout the hydrogel matrix. The presence of pore structures and protruding nanofibers at fracture surfaces indicates that failure primarily occurred within the CS/SA matrix through a fiber pull-out mechanism. The reinforcement mechanism of PMIA nanofibers in hydrogel matrices primarily involves two key aspects: (i) load transfer from the weaker matrix to the higher-strength nanofibers under external stress; (ii) crack propagation inhibition through nanofiber-induced obstruction.

### 3.2. Mechanical Properties and Structure of Meta-Aramid Nanofiber-Reinforced Composite Hydrogel

The PMIA-SA/CS composite hydrogel was prepared through a reaction–diffusion approach. Specifically, a homogeneous mixture of PMIA nanofibers and sodium alginate (SA) was uniformly coated onto a glass substrate. The coated substrate was then immersed in a low-molecular-weight chitosan (CS) solution. During this process, CS molecules electrostatically complex with SA, forming a dense CS/SA complexation layer at the solution interface. Subsequently, CS molecules progressively penetrated this complexation layer through diffusion, continuously reacting with the inner SA via complexation. This diffusion-controlled reaction led to a gradual increase in the thickness of the complexation layer. Ultimately, a PMIA-SA/CS composite hydrogel with a three-dimensional network structure was successfully fabricated through this reaction-diffusion process.

**Figure 2:**
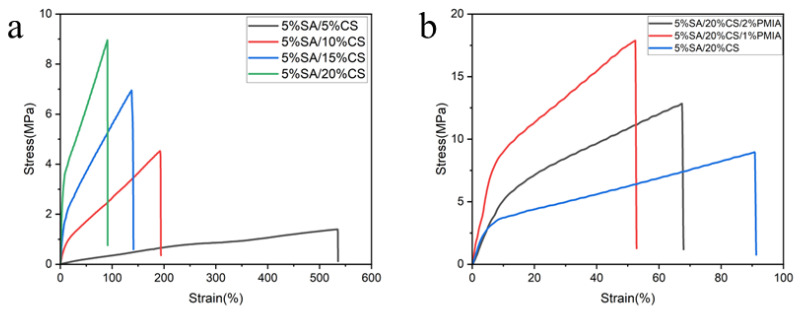
(**a**) Stress–strain curves of the SA/CS hydrogels prepared with different concentrations of CS. (**b**) Stress–strain curves of PMIA-SA/CS composite hydrogels prepared with different concentrations of PMIA nanofibers.

To construct high-strength composite hydrogels, we first optimized the chitosan (CS)/sodium alginate (SA) hydrogel matrix, as shown in Figure 2a and Table 2. Figure 2a presents the mechanical properties of CS/SA hydrogels prepared with 5%SA and varying CS concentrations. The results demonstrate that increasing CS concentration leads to gradual enhancement in tensile strength but reduction in elongation at break. This phenomenon originates from the elevated electrostatic complexation density within the hydrogel network, which increases the physical crosslinking density (i.e., sacrificial bond density) and consequently improves tensile strength. However, the higher crosslinking density simultaneously restricts polymer chain mobility and reduces the maximum extensibility between crosslinks, ultimately decreasing the fracture strain.

**Figure 3:**
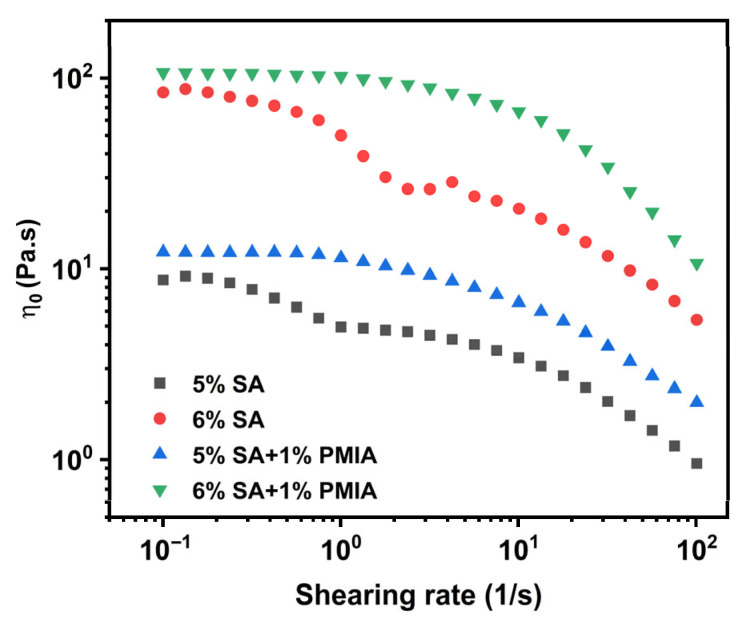
Viscosity of the SA/CS hydrogels prepared with different concentrations of PMIA.

Then, PMIA nanofibers were incorporated into the sodium alginate (SA) matrix. While increasing SA concentration generally facilitates the formation of high-strength composite gels, we observed that at elevated SA concentrations, the introduction of PMIA nanofibers significantly increased the viscosity of the mixed solution (as shown in Figure 3), resulting in poor homogeneity and substantial processing challenges. Based on our experimental optimization, the concentrations of SA and chitosan (CS) were fixed at 5 wt% and 20 wt%, respectively. By introducing 1 wt% and 2 wt% PMIA nanofibers into the SA solution, we successfully prepared PMIA-SA/CS composite hydrogels with varying reinforcement levels.

To validate the reinforcing effect of PMIA nanofibers on the hydrogel matrix, we characterized the mechanical properties of PMIA-SA/CS hydrogels incorporated with varying PMIA nanofiber concentrations (as shown in Figure 2b). The results demonstrate that the incorporation of PMIA nanofibers significantly enhances the tensile strength of the composite hydrogels. The tensile strength attains its maximum value at 1 wt% loading. Conversely, the fracture strain shows an opposite trend, attaining its minimum value at 1 wt%. The optimal mechanical performance is achieved at 1 wt% PMIA loading, with the fracture stress of 16.8 MPa and fracture strain of 48%, which is better than other hydrogels reported in the literature (as shown in Table 3). However, excessive PMIA content (excess 2 wt%) leads to the formation of micron-scale aggregates due to uneven dispersion, which act as stress concentrators and consequently degrade the mechanical properties. Notably, the composite hydrogel with 1 wt% PMIA exhibits a distinct yield phenomenon, indicating strong interfacial interactions (e.g., π-π stacking and hydrogen bonding) between the nanofibers and hydrogel matrix. These interactions effectively restrict the segmental motion of polymer chains within the PMIA-SA/CS network, resulting in forced elastic deformation under external stress and consequently manifesting the yield behavior.

**Figure 4:**
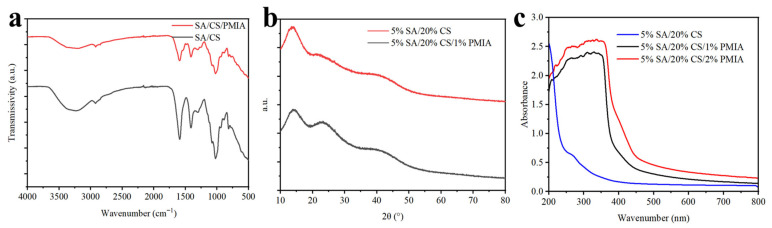
(**a**) The Fourier transform infrared spectra of the SA/CS hydrogel membrane and PMIA-SA/CS composite hydrogel membrane. (**b**) XRD spectra of the SA/CS hydrogel membrane and PMIA-SA/CS composite hydrogel membrane. (**c**) The UV-visible spectra of the SA/CS hydr.

The FTIR spectra of SA/CS and PMIA-SA/CS hydrogel are shown in Figure 4a. The peak at 1070–1150 cm^−1^ is the stretching vibration absorption peak of the pyranose ring ether bond (C-O-C), which exists in both SA and CS. The peak at 1425 cm^−1^ corresponds to the symmetric stretching vibration of the carboxylate group (-COO^−^). The peak at 1631 cm^−1^ corresponds to the C=O stretching vibration of the amide group in CS and -O-H bending vibration of hydroxyl in both SA and CS. The peak at 2926 cm^−1^ is the stretching vibration of methylene (-CH_2_-). The peak at 3200–3400 cm^−1^ is the stretching vibration of hydroxyl and amino. The introduction of nanofibers had almost no effect on the FTIR spectrum of the SA/CS hydrogel, likely because the nanofiber content was too low and its absorption peaks were masked by SA/CS.

The XRD patterns in Figure 4b demonstrate that the incorporation of PMIA nanofibers induces significant crystalline modifications in the SA/CS hydrogel matrix. The weakened diffraction peaks between 10 and 20° suggest that PMIA nanofibers restrict crystal growth along these specific lattice planes by disrupting the original molecular packing and introducing structural defects, leading to reduced crystallinity in this angular range. Conversely, the enhanced peak intensity observed between 20 and 30° indicates that PMIA nanofibers enhanced local ordering at the interface that promotes molecular chain alignment and increases local crystallinity through epitaxial growth along these preferred crystallographic orientations [25]. This dual effect of crystalline reorganization, involving both suppression of certain crystal planes and enhancement of others, creates an optimized microstructure that contributes substantially to the composite hydrogel’s exceptional mechanical performance. The precisely controlled crystalline domains facilitate efficient stress transfer, inhibit molecular chain slippage, and improve energy dissipation capacity, collectively explaining the remarkable tensile strength observed in PMIA-SA/CS composite hydrogels. These XRD findings provide direct structural evidence for the reinforcement mechanism at the molecular level, complementing the mechanical property measurements and microscopic observations presented earlier.

**Table 3:** Mechanical properties of different nano-reinforced composite hydrogels.

Sample	Young’s Modulus (MPa)	Max Tensile Strength (MPa)	Max Elongation at Break (%)
Hydrogel Reinforced by Meta-Aramid Nanofibers	34.420	17.900	52
optimized laminated hydrogel/fiber composite [26]	2.180	0.129	5.9
tough NC organo-hydrogels [27]	0.050	0.178	356.17
ANF Reinforced PVA Hydrogels [28]	15.400	5.500	34
Hydrogels Dual-Reinforced by Cellulose [29]	0.002	0.056	3000

Due to the extensive π-π interactions within aramid nanofibers, they exhibit strong ultraviolet (UV) absorption capacity. Therefore, the incorporation of aramid nanofibers significantly reduces the UV transmittance of PMIA-SA/CS hydrogels, as shown in Figure 4c. Additionally, the SA/CS film itself possesses good optical transparency. However, PMIA, as a filler, introduces microscopic defects (such as interfacial voids and crystalline regions), leading to increased light scattering and thereby reducing the visible light transmittance of the PMIA-SA/CS hydrogel film. Owing to this exceptional UV absorption capability, the PMIA-SA/CS hydrogel demonstrates great potential for applications in UV protection and related fields.

### 3.3. Sensing Performance of Hydrogel Sensor Reinforced by Meta-Aramid Nanofiber

**Figure 5:**
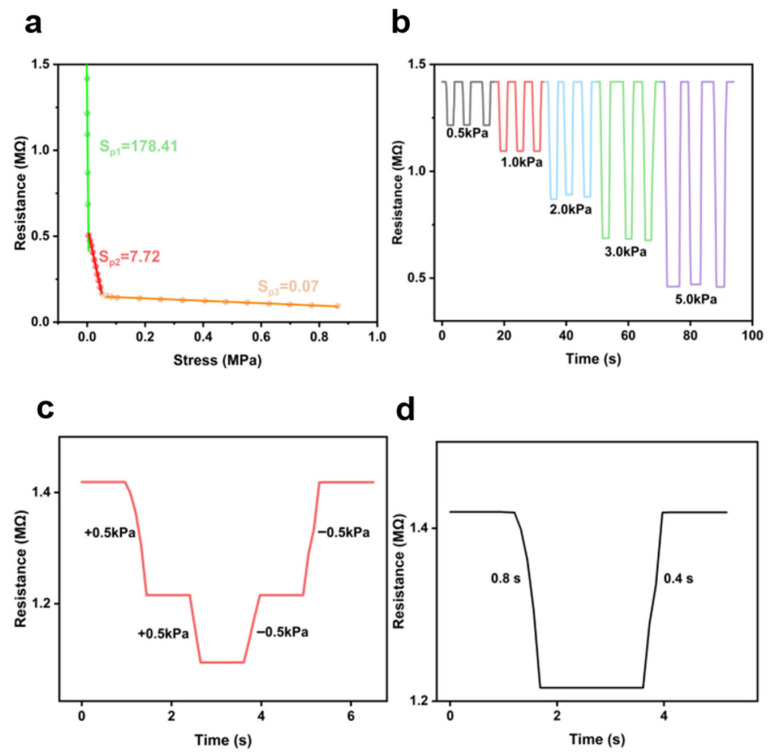
Resistance of PMIA-SA/CS composite hydrogels in response to different pressure: (**a**) gradually increased pressure; (**b**) repeated loading and unloading of 0.5 kPa–5.0 kPa pressures; (**c**) the gradual loading and removing of 0.5 kPa pressure; (**d**) the loading a.

Benefiting from the abundant ionizable groups within the PMIA-SA/CS composite hydrogel, it exhibits excellent ionic conductivity, making it suitable for use as an ion-based pressure sensor. Figure 5a shows the resistance variation curve of the PMIA-SA/CS hydrogel-based pressure sensor during continuous compression. When pressure is applied, the thickness of the SA-PMIA/CS hydrogel decreases while its cross-sectional area increases, leading to a reduction in resistance. Here, we introduce pressure sensitivity (Sp = ΔR/ΔP) to characterize the pressure-sensing performance of the composite hydrogel sensor, where ΔR and ΔP represent changes in resistance and pressure, respectively. At pressures below 5 kPa, the PMIA-SA/CS hydrogel-based pressure sensor demonstrates a high sensitivity of 178.41 MΩ/MPa. However, as the pressure increases, the sensitivity of the SA-PMIA/CS film decreases—dropping to 7.72 MΩ/MPa in the 5–50 kPa range and further declining to 70 kΩ/MPa at pressures exceeding 50 kPa. This behavior can be attributed to the increased modulus of the hydrogel material under compression, which reduces its deformation rate and consequently lowers sensing sensitivity.

The pressure-sensing performance of the SA-PMIA/CS hydrogel sensor was evaluated by applying repeated pressures of varying magnitudes, as illustrated in Figure 5b. The results demonstrate that the PMIA-SA/CS hydrogel sensor can accurately detect subtle pressures as low as 0.5 kPa and generate distinct resistance signal responses corresponding to different pressure levels. During three consecutive test cycles within the 0.5–5 kPa range, the sensor exhibited highly reproducible waveforms, confirming its excellent sensing stability.

Furthermore, the SA-PMIA/CS hydrogel sensor exhibited precise detection of static pressure stimuli, with rapid response upon both pressure application and release (Figure 5c,d). When a 0.5 kPa pressure was applied, the resistance immediately decreased and stabilized. Upon an additional 0.5 kPa pressure increment, the SA-PMIA/CS film maintained its rapid responsiveness, displaying a stepwise response curve. Similarly, during gradual pressure release, the resistance swiftly recovered to its initial baseline (Figure 5c). Notably, the SA-PMIA/CS hydrogel pressure sensor demonstrated an ultrafast response time of approximately 0.4–0.8 s (Figure 5d).

**Figure 6:**
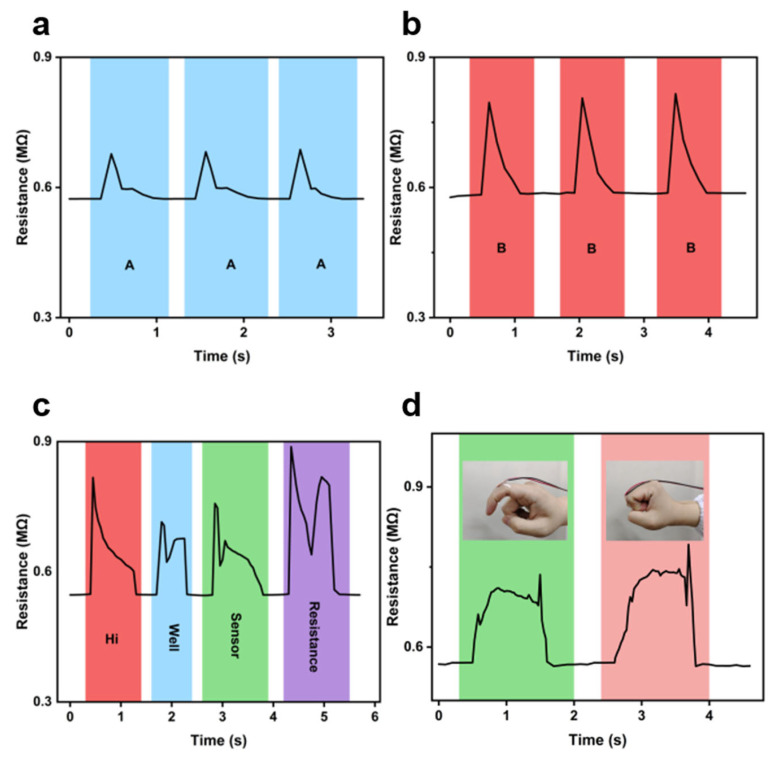
Resistance of PMIA-SA/CS composite hydrogels in response to different human motions: (**a**–**c**) vocal cord vibration when speaking letters of “A”, “B” and other words; (**d**) different bending degrees of finger.

To investigate the sensing performance of the SA-PMIA/CS film in detecting subtle pressures, a simple sensing device was fabricated using the PMIA-SA/CS composite hydrogel membrane. The device was attached to the throat and the second knuckle of the right index finger to monitor resistance changes during different human activities, as shown in Figure 6. The results revealed that the PMIA-SA/CS hydrogel sensor could effectively detect vocal cord vibrations during speech, generating distinct waveform patterns for different pronunciations. Remarkably, it produced identical waveforms when the same words were repeated (Figure 6a–c). Additionally, the sensor exhibited responsive signals to varying degrees of finger bending (Figure 6d). These findings demonstrate the significant potential of the SA-PMIA/CS membrane for applications in medical diagnostics, health monitoring, and motion tracking.

**Figure 7:**
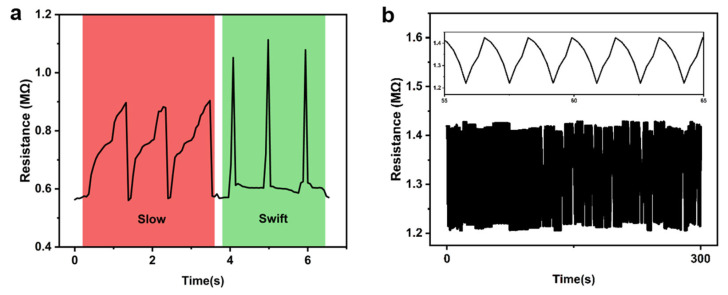
(**a**) Resistance of PMIA-SA/CS composite hydrogels in response to slow and swift finger compression. (**b**) Resistance signal of PMIA-SA/CS composite hydrogels in response to cyclic loading/unloading of a compression stress.

To further evaluate the practical application potential of the PMIA-SA/CS hydrogel sensor, we simulated real-world usage scenarios by attaching the sensor to a rubber glove to monitor electrical signal variations during typing (Figure 7a). As evident from Figure 7a, the sensor exhibited distinct signal patterns corresponding to different typing motions: a step-like electrical signal was observed during slow key presses, while rapid keystrokes generated sharp peak-shaped signals. Additionally, we also test the long-term stability of the PMIA-SA/CS composite hydrogel sensor, as shown in Figure 7b. It shows an outstanding long-term stability with no signal attenuation observed even after 200 compression cycles.

## 4. Conclusions

In this study, we successfully developed ultra-high-strength meta-aramid (PMIA) nanofiber-reinforced SA/CS composite hydrogels through a diffusion–complexation strategy coupled with nanocomposite reinforcement. The resulting hydrogel exhibited exceptional mechanical properties, achieving a tensile strength of 16.8 MPa—far surpassing conventional polysaccharide-based hydrogels—while maintaining good biocompatibility and processability. Structural characterization confirmed that the PMIA nanofibers formed an interpenetrating network within the SA/CS matrix, enhancing stress transfer and energy dissipation through interfacial hydrogen bonding and π-π interactions. The optimized hydrogel demonstrated outstanding pressure-sensing performance, including high sensitivity (178.41 MΩ/MPa at <5 kPa), rapid response (0.4–0.8 s), and excellent stability over 200 cycles. Practical applications in health monitoring (e.g., vocal cord vibration detection and finger motion tracking) highlighted its potential for wearable sensors and soft robotics. This work provides a scalable strategy for designing natural polysaccharide-based hydrogels with synthetic-polymer-level performance, bridging the gap between mechanical robustness and biological compatibility for next-generation flexible devices. Future research should explore long-term in vivo stability and signal precision in dynamic physiological environments.

## Figures and Tables

**Table 1:** Formulation of samples.

Sample	SA	CS	PMIA
5% SA + 5%CS	5%	5%	
5% SA + 10% CS	5%	10%	
5% SA + 15% CS	5%	15%	
5% SA + 20% CS	5%	20%	
6% SA + 20% CS	6%	20%	
5% SA + 20% CS + 1% PMIA	5%	20%	1%
5% SA + 20% CS + 2% PMIA	5%	20%	2%
6% SA + 20% CS + 1% PMIA	6%	20%	1%

**Table 2:** Comparison of mechanical properties of hydrogels with different formulations.

Sample	Young’s Modulus (MPa)	Max Tensile Strength (MPa)	Max Elongation at Break (%)	Toughness (J/m^3^)
5% SA + 5% CS	0.243	1.91	786	412.7
5% SA + 10% CS	2.34	4.51	193	502.3
5% SA + 15% CS	5.02	6.94	138	613.0
5% SA + 20% CS	7.40	8.96	121	536.7
5% SA + 20% CS + 1% PMIA	34.42	17.9	52	637.4
5% SA + 20% CS + 2% PMIA	19.10	12.8	67	571.8

## Data Availability

Data is contained within the article.

## Abbreviations

The following abbreviations are used in this manuscript:

SA	Alginate
CS	Chitosan
DMAc	Dimethylacetamide
PMIA	meta-Aramid
XRD	X-Ray Diffraction
SEM	Scanning Electron Microscope
TEM	Transmission Electron Microscope
